# Effects of Coloring Food Images on the Propensity to Eat: A Placebo Approach With Color Suggestions

**DOI:** 10.3389/fpsyg.2020.589826

**Published:** 2020-10-29

**Authors:** Carina Schlintl, Anne Schienle

**Affiliations:** Department of Clinical Psychology, University of Graz, Graz, Austria

**Keywords:** food coloring, colored backgrounds, blue, red, verbal suggestions, placebo, food wanting

## Abstract

**Background:**

Research findings on the appetite-enhancing effect of the color red and the appetite-reducing effect of blue have been inconsistent. The present study used a placebo approach and investigated whether verbal suggestions can enhance color-appetite effects.

**Method:**

A total of 448 women participated in two experiments. They viewed images with differently colored sweet foods (original color, blue, red, colorless (black-and-white); experiment 1; *n* = 217) or sweet foods on blue, red, white, and gray backgrounds; experiment 2; *n* = 231). Before viewing the images, half of the participants received information about the effects of red and blue food color on appetite (color suggestion). The other half received no suggestion. For each of the experiments, the reported propensity to eat (food wanting) was compared between the conditions.

**Results:**

All colored food items were associated with a lower propensity to eat compared to the food items in the original color. The color suggestion (compared to no suggestion) additionally decreased the propensity to eat blue and black-and-white food items. Colored backgrounds did not influence food wanting.

**Conclusion:**

This study demonstrated that red and blue coloring of visual food cues did not have the predicted effects on food wanting. However, the combination of specific food colors with specific color suggestions might be useful to change the willingness to eat sweet products.

## Introduction

Color signals the edibility and the nutritional value of food (Spence, 2015). The food color red is very common in nature and is typical for ripe fruits and fresh meat. In contrast, there aren’t many naturally occurring blue-hued foods, and sometimes ‘blue’ even indicates non-edibility (e.g., mold). Therefore, it is not surprising that the color red is considered appetizing, whereas blue acts as an appetite suppressant (for reviews see Spence et al., 2010; Zellner, 2013; Wadhera and Capaldi-Phillips, 2014; Spence, 2015).

Based on these observations, the adding of coloring to food and drink has a long history in the food industry. However, research on the effects of red/blue food coloring on the wanting and liking of food has produced heterogeneous results. In some of the studies the predicted effects occurred (e.g., increased appetite for red-colored food: e.g., Foroni et al., 2016, decreased appetite for blue-colored food; e.g., Cho et al., 2015; Suzuki et al., 2017), but not in other studies (e.g., Gifford et al., 1987; Frank et al., 1989, Chan and Kane-Martinelli, 1997; Alley and Alley, 1998).

Discrepant findings also characterize the research on the influence of color context (e.g., color of dishware) on food wanting, liking, and consumption (e.g., Tomita et al., 2007; Genschow et al., 2012, Piqueras-Fiszman et al., 2012; for a review see Spence, 2018). For example, some studies have shown that red plates/cups increase appetite and food/drink consumption (Piqueras-Fiszman and Spence, 2012), whereas other investigations even reported the opposite effect (e.g., Genschow et al., 2012).

The color of food not only provides information about the edibility but also about the palatability of food (Spence, 2015). The evaluation of the hedonic reward from food is highly susceptible to suggestion (Shankar et al., 2010). Placebo research has shown that the desire to eat specific food items can be influenced by verbal suggestions (e.g., Crum et al., 2011; Hoffmann et al., 2018; Potthoff et al., 2019). For example, participants consumed less in a test session when they were reminded of their last meal (Higgs, 2002), when the food was labeled ‘healthy’ (Provencher and Jacob, 2016) or ‘high-caloric’ (Crum et al., 2011). In a study by Schienle et al. (2020), a placebo (inert treatment of the tongue) was able to alter taste sensations and the affective ratings for food pictures. Pictures of spoiled food (with black/blue mold) were rated as less disgusting in the placebo condition (compared to the condition without placebo). Thus, the wanting and liking of food (cues) can be shaped by inducing expectations through verbal suggestions.

The aim of the present study was twofold. First, we attempted to replicate the effects of red/blue food coloring and red/blue backgrounds on food wanting (i.e., the propensity to eat). A large sample (*n* = 448) was examined to overcome problems of previous studies on food-color effects with small sample sizes. Second, we attempted to enhance the color-appetite effects by using verbal suggestions. The participants viewed images with colored food items (original color, blue, red, black-and-white) or food items on colored backgrounds (white, blue, red, gray). Before viewing the pictures, half of the participants received the information that red color increases appetite, and blue color acts as an appetite suppressant (color suggestion); the other half received no suggestion. Ratings for food wanting (the propensity to eat the depicted food item) were compared between the conditions in each of the experiments.

## Materials and Methods

### Participants

A total of 448 females aged between 18 and 35 years (*M* = 22.54 years; *SD* = 3.36) participated in two experiments (experiment 1: *n* = 217; experiment 2: *n* = 231). The participants had a mean body mass index (BMI) of *M* = 21.74 (*SD* = 2.92). The reported hunger level at the time of testing was *M* = 3.04 (*SD* = 2.26; 1 = not hungry; 9 = very hungry), and the average time since the last meal was *M* = 3.30 h (*SD* = 3.53). We only tested females because of reported sex differences concerning self-reports for appetite and food preferences (e.g., Blechert et al., 2014; Gregersen et al., 2011, Bédard et al., 2015). Participants were recruited via announcements at the university campus; the majority were students (91%).

The computed pairwise comparisons (*t*-tests) did not show statistically significant differences between the participants assigned to the two conditions (suggestion vs. no-suggestion) in each of the two experiments concerning BMI, hunger level, and time since last meal (all *p* > 0.19; for means (*M*) and standard deviations (*SD*) see Supplementary Table 1).

### Stimuli and Design

The stimulus material for experiment 1 consisted of 12 images of sweet foods (e.g., chocolate chip cookie, cupcake, and cream cake) taken from the Food Pics Database (Blechert et al., 2014) and non-copyrighted sources from the internet. We selected images of sweet food because these stimuli receive on average neutral to positive ratings for food wanting (Blechert et al., 2014). Thus, changes in reported food wanting can be induced, including both an increase and a decrease.

**FIGURE 1:**
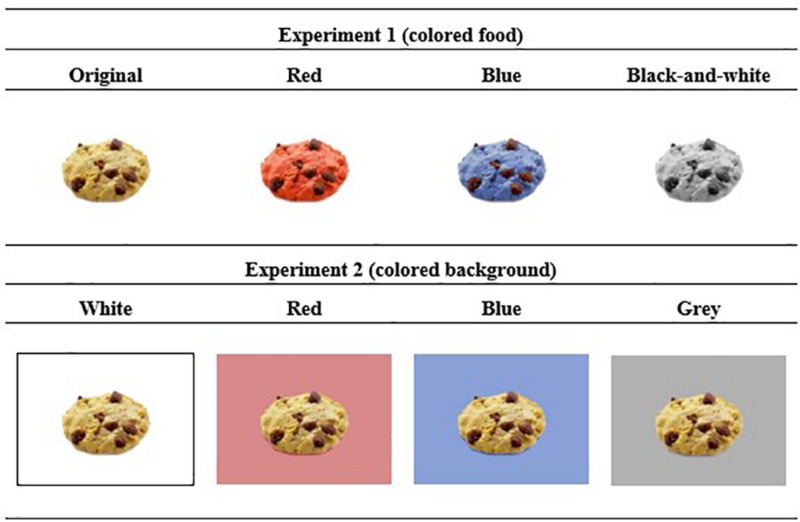
Examples of food images in the two experiments.

For each of the 12 original pictures, three additional versions with the food items colored in blue, red, and black-and-white (colorless) were created (see Figure 1), resulting in a total of 48 food images. The black-and-white images served as a control condition that was characterized by the absence of blue and red color. The original images were considered the reference or baseline condition (reflecting individual preferences concerning a food item). The images had a resolution of either 600 × 450 pixels or 350 × 500 pixels. Luminance scores for blue and red color were equivalent (160 lm).

In Experiment 2, the same 12 food images were used as in experiment 1 (in original color). Four different versions were created with a white, red, blue, and gray (black-and-white) background (see Figure 1). In both experiments, the same red and blue coloring was used.

### Procedure

The images of experiment 1 and experiment 2 were presented via two independent online surveys (LimeSurvey GmbH, Hamburg). To avoid boredom and habituation because of the repeated presentation of the same food images in different color versions, each participant was presented with a random selection of 12 pictures (three images in blue, red, black-and-white (gray), and original color). The pictures were displayed in randomized order and the participants rated their food wanting (“How much would you like to eat this food right now?”) on a 7-point Likert scale (1 = not at all; 7 = very much) for each picture.

In both experiments, the participants were randomly assigned to one of two experimental conditions. In the color suggestion condition, the participants were provided with information about the appetizing effect of the color red and the appetite-suppressant effect of blue. Participants of the no suggestion condition received no color information.

The study was approved by the ethics committee of the University and was performed following the Declaration of Helsinki. All participants gave written informed consent.

### Statistical Analysis

To adjust for individual differences in food wanting for the original items (*M* = 3.87; *SD* = 1.56; range = 1–7), we computed difference scores. To do this, we first calculated mean scores for each color condition based on the three ratings for wanting of each participant. Then, difference scores (between the conditions) were calculated (Experiment 1: Difference score_red: wanting for red-colored food minus wanting for food in the original color, Difference score_blue: blue minus original, Difference score_black-and-white: black-and-white minus original; Experiment 2: Difference score_red: wanting for food on red background minus wanting for food on white background, Difference score _blue: blue minus white, Difference score_gray: gray minus white).

For each of the two experiments, a 3 × 2 analysis of variance (ANOVAs) was performed to test the effects of COLOR (Difference score_red, Difference score _blue, Difference score _black-and-white/grey) and CONDITION (color suggestion, no-suggestion) on the propensity to eat. After controlling for hunger level, BMI, and hours since the last meal in additionally computed analyses of covariance (ANCOVA), the results did not change. Therefore, we report the ANOVA findings.

Effect sizes are expressed by partial eta squared (*part.*η*^2^*). If violations of sphericity occurred, Greenhouse-Geisser corrections were used. Significant effects were followed up by Bonferroni-adjusted pairwise comparisons. The analyses were conducted with SPSS version 26 (IBM Corp, 2019).

A power analysis with G^∗^Power 3.1.9.2 Faul et al. (2007) indicated that a sample size of *n* = 192 would be necessary to detect an effect size of *part.*η*^2^* = 0.03 (i.e., small effect) with a probability of 1–β = 0.80, α = 0.05 for the interaction effect COLOR x CONDITION.

## Results

### Experiment 1 (Colored Food)

**FIGURE 2:**
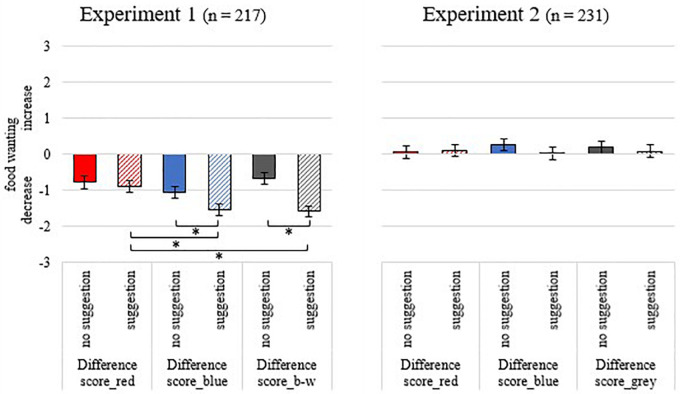
Means and standard errors of difference scores for food wanting in experiment 1 and experiment 2 across the different conditions. Experiment 1: “Difference score_red” = wanting of red-colored food items minus food in original color; “Difference score_blue” = wanting of blue-colored food minus food in original color; “Difference score_b-w” = wanting of black-and-white colored food minus food items in original color; Experiment 2: “Difference score_red” = wanting of food on red background minus white background; “Difference score_blue” = wanting of food on blue background minus white background; “Difference score_gray” = wanting of food on grey background minus white background; Asterisks (^∗^) indicate *p* < 0.05.

The ANOVA revealed significant effects for COLOR [*F*(2, 430) = 8.74, *p* < 0.001, *part.*η*^2^* = 0.039], COLOR x CONDITION [*F*(2, 430) = 6.34, *p* = 0.002, *part.*η*^2^* = 0.029] and CONDITION [*F*(1, 215) = 6.41, *p* = 0.012, *part.*η*^2^* = 0.029]. Compared to original color, the coloring (red, blue, black-and-white) of the food items reduced the propensity to eat significantly (all *p* < 0.001). The reduction in food wanting was larger for blue and black-and-white food in the suggestion condition than in the no-suggestion condition (all *p* < 0.047; see Figure 2). The ratings for red food did not differ between the suggestion and no-suggestion condition (*p* = 0.653).

In the suggestion condition, the reduction in the propensity to eat blue and black-and-white food was greater compared to red food (all *p* < 0.001). Blue and black-and-white food did not differ from each other (*p* > 0.99). In the no-suggestion condition, food wanting did not differ between the color conditions (all *p* > 0.072; Figure 2).

### Experiment 2 (Colored Backgrounds)

The ANOVA revealed no statistically significant results for COLOR [*F*(1.93, 442.36) = 0.204, *p* = 0.816, part.η*^2^* = 0.001], CONDITION [*F*(1, 229) = 0.261, *p* = 0.610, *part.*η*^2^* = 0.001], and the interaction COLOR x CONDITION [*F*(1.93, 442.36) = 1.079, *p* = 0.339, *part.*η*^2^* = 0.005; Figure 2]. Food wanting did not differ between food items with white backgrounds and food with colored backgrounds (red, blue, gray; all *p* > 0.243).

## Discussion

This study examined the effects of red/blue coloring of visual food cues and verbal color suggestions on reported food wanting. It was shown that both blue and red coloring of the depicted food items had an appetite-reducing effect. Thus, ‘red’ and ‘blue’ did not have the predicted opposite effects on the propensity to eat but were always considered negative. For example, compared to the original brown chocolate chip cookie, all color variants (red, blue, black-and-white) were experienced as less appetizing. In the no-suggestion condition, the appetite-reducing effect of ‘blue’ and ‘red’ did not differ from each other. This effect very likely is a result of ‘color expectancy deviations’. We all have concepts of how specific food items should look like. If a ‘color expectancy violation’ occurs, this induces reductions in food wanting. In a classic study by Wheately (1973), participants were presented with a dinner consisting of a blue steak, red peas, and green French fries. The dinner started under dim lighting to hide the food’s true color. When the lighting was returned to normal, the ‘inappropriate’ food coloring elicited appetite reduction and even nausea in some of the participants. In a recent study by Suzuki et al. (2017), blue soup decreased reported appetite and palatability compared to soup with typical colors (white, yellow).

In the present investigation, the ‘blue effect’ on reported food wanting was enhanced by the verbal suggestion of this color as an appetite suppressant. Additionally, we observed an appetite-reducing effect of black-and-white coloring in the suggestion condition. An explanation might be that achromatic/black-and-white is more likely perceived as belonging to the blue color spectrum (e.g., Weiss et al., 2017) and that toxic or spoiled food is often blue, black, or purple. The ‘red suggestion’ did not affect food wanting because an appetite increase was suggested, while the participants experienced a reduction.

No influence of color on food wanting was observed in experiment 2 although our large sample size was associated with sufficient power to detect even small effects. Previous studies have reported effects of different color contexts, such as table cloths, plates, cups, and ambient illumination on appetite and taste ratings (for a review see Spence, 2018). A possible explanation for the absence of the color-background effect in the present experiment can be derived from the findings by Schifferstein et al. (2016). The authors presented five differently colored vegetables (tomato, carrot, yellow bell pepper, cucumber, and eggplant) against one of four different backgrounds (either light or dark orange or light or dark blue). The participants rated the attractiveness of the vegetables. The main result of this study was that each food item had its optimal background color. For example, a light orange made the cucumber most attractive, while light blue was optimal for the eggplant. Thus, different food items seem to be associated with different appetizing contexts.

The following limitations of the current study need to be addressed. In the present study, female participants (mainly university students) were presented with images depicting sweet foods. Thus, our results cannot be generalized to other samples and food types. Moreover, the coloring of images vs. real food items might have different effects. Therefore, in a future study, the consumption of colored food items (e.g., amount of food eaten in a test meal) should be assessed. The present study only relied on self-reports for the propensity to eat the depicted food items.

## Conclusion

In conclusion, this study identified conditions under which color suggestions can influence food wanting. Future research now needs to find optimal combinations of food coloring and color suggestions for specific food items to alter the propensity to eat in the intended direction.

## Data Availability Statement

The raw data supporting the conclusions of this article will be made available by the authors, without undue reservation.

## Ethics Statement

The studies involving human participants were reviewed and approved by Ethics committee of the University of Graz. The patients/participants provided their written informed consent to participate in this study.

## Author Contributions

CS and AS designed the study. CS collected and analyzed the data. AS wrote the manuscript. Both authors contributed to the article and approved the submitted version.

## Conflict of Interest

The authors declare that the research was conducted in the absence of any commercial or financial relationships that could be construed as a potential conflict of interest.

## Supplementary Material

Supplementary Table 1 Descriptive Statistics (means and standard deviations (M, SD) for the participants in the non-suggestion condition and the suggestion condition in Experiment 1 and Experiment 2.

## References

[B1] Alley R. L. Alley T. R. (1998). The influence of physical state and color on perceived sweetness. *J. Psychol.* 132 561–568. 10.1080/00223989809599289 9729847

[B2] Bédard A. Hudon A. M. Drapeau V. Corneau L. Dodin S. Lemieux S. (2015). Gender differences in the appetite response to a satiating diet. *J. Obesity* 2015 1–9. 10.1155/2015/140139 26442158 PMC4579320

[B3] Blechert J. Meule A. Busch N. A. Ohla K. (2014). Food-pics: an image database for experimental research on eating and appetite. *Front. Psychol.* 5:617. 10.3389/fpsyg.2014.00617 25009514 PMC4067906

[B4] Chan M. M. Kane-Martinelli C. (1997). The effect of color on perceived flavor intensity and acceptance of foods by young adults and elderly adults. *J. Am. Diet. Assoc.* 97 657–659. 10.1016/s0002-8223(97)00165-x 9183329

[B5] Cho S. Han A. Taylor M. H. Huck A. C. Mishler A. M. Mattal K. L. (2015). Blue lighting decreases the amount of food consumed in men, but not in women. *Appetite* 85 111–117. 10.1016/j.appet.2014.11.020 25447013

[B6] Crum A. J. Corbin W. R. Brownell K. D. Salovey P. (2011). Mind over milkshakes: mindsets, not just nutrients, determine ghrelin response. *Health Psychol.* 30 424–429. 10.1037/a0023467 21574706

[B7] Faul F. Erdfelder E. Lang A. -G. Buchner A. (2007). G^∗^power 3: a flexible statistical power analysis program for the social, behavioral, and biomedical sciences. *Behav. Res. Methods* 39 175–191. 10.3758/BF03193146 17695343

[B8] Foroni F. Pergola G. Rumiati R. I. (2016). Food color is in the eye of the beholder: the role of human trichromatic vision in food evaluation. *Sci. Rep.* 6:37034. 10.1038/srep37034 PMC5107980 27841327

[B9] Frank R. Ducheny K. Mize S. (1989). Strawberry odor, but not red color, enhances the sweetness of sucrose solutions. *Chem. Senses* 14 371–377. 10.1093/chemse/14.3.371

[B10] Genschow O. Reutner L. Wänke M. (2012). The color red reduces snack food and soft drink intake. *Appetite* 58 699–702. 10.1016/j.appet.2011.12.023 22245725

[B11] Gifford S. R. Clydesdale F. M. Damon R. A. Jr. (1987). The psychophysical relationship between color and salt concentrations in chicken flavored broths. *J. Sens. Stud.* 2 137–147. 10.1111/j.1745-459x.1987.tb00193.x

[B12] Gregersen N. Møller B. Raben A. Kristensen S. Holm L. Flint A. (2011). Determinants of appetite ratings: the role of age, gender, BMI, physical activity, smoking habits, and diet/weight concern. *Food Nutr. Res.* 55:7028. 10.3402/fnr.v55i0.7028 21866221 PMC3160809

[B13] Higgs S. (2002). Memory for recent eating and its influence on subsequent food intake. *Appetite* 39 159–166. 10.1006/appe.2002.0500 12354684

[B14] Hoffmann V. Lanz M. Mackert J. Müller T. Tschöp M. Meissner K. (2018). Effects of placebo interventions on subjective and objective markers of appetite–a randomized controlled trial. *Front. Psychiatry* 9:706. 10.3389/fpsyt.2018.00706 30618877 PMC6305288

[B15] IBM Corp. (2019). *IBM SPSS Statistics for Windows*, Version 26 Armonk, NY: IBM Corp.

[B16] Piqueras-Fiszman B. Spence C. (2012). The influence of the color of the cup on consumers’ perception of a hot beverage. *J. Sensory Stud.* 27 324–331. 10.1111/j.1745-459X.2012.00397.x

[B17] Piqueras-Fiszman B. Alcaide J. Roura E. Spence C. (2012). Is it the plate or is it the food? Assessing the influence of the color (black or white) and shape of the plate on the perception of the food placed on it. *Food Qual. Prefer.* 24 205–208. 10.1016/j.foodqual.2011.08.011

[B18] Potthoff J. Jurinec N. Schienle A. (2019). Placebo effects on visual food cue reactivity: an eye-tracking investigation. *Front. Psychiatry* 10:525. 10.3389/fpsyt.2019.00525 31396116 PMC6667658

[B19] Provencher V. Jacob R. (2016). Impact of perceived healthiness of food on food choices and intake. *Curr. Obesity Rep.* 5 65–71. 10.1007/s13679-016-0192-0 26820622

[B20] Schienle A. Gremsl A. Schwab D. (2020). Placebos can change affective contexts: an event-related potential study. *Biol. Psychol.* 150:107843. 10.1016/j.biopsycho.2020.107843 31945399

[B21] Schifferstein H. N. J. Howell B. F. Pont S. (2016). Colored backgrounds affect the attractiveness of fresh produce, but not it’s perceived color. *Food Qual. Prefer.* 56 173–180. 10.1016/j.foodqual.2016.10.011

[B22] Shankar M. U. Levitan C. A. Spence C. (2010). Grape expectations: the role of cognitive influences in color–flavor interactions. *Conscious. Cogn.* 19 380–390. 10.1016/j.concog.2009.08.008 19828330

[B23] Spence C. (2015). On the psychological impact of food colour. *Flavour* 4:21 10.1186/s13411-015-0031-3

[B24] Spence C. (2018). Background colour & its impact on food perception & behaviour. *Food Qual. Prefer.* 68 156–166.

[B25] Spence C. Levitan C. A. Shankar M. U. Zampini M. (2010). Does food color influence taste and flavor perception in humans? *Chemosens. Percept.* 3 68–84. 10.1007/s12078-010-9067-z

[B26] Suzuki M. Kimura R. Kido Y. Inoue T. Moritani T. Nagai N. (2017). Color of hot soup modulates postprandial satiety, thermal sensation, and body temperature in young women. *Appetite* 114 209–216. 10.1016/j.appet.2017.03.041 28373021

[B27] Tomita K. Ono M. Aiba T. Ohtani K. (2007). Psychological effects of tablecloth color on diners under different brightness. *Jpn. Assoc. Integr. Study Dietary Habits* 18 48–55. 10.2740/jisdh.18.48 17326625

[B28] Wadhera D. Capaldi-Phillips E. D. (2014). A review of visual cues associated with food on food acceptance and consumption. *Eat. Behav.* 15 132–143. 10.1016/j.eatbeh.2013.11.003 24411766

[B29] Weiss D. Witzel C. Gegenfurtner K. (2017). Determinants of colour constancy and the blue bias. *i Percept.* 8 1–29. 10.1177/2041669517739635 PMC5768282 29348910

[B30] Wheately J. (1973). Putting color into marketing. *Marketing* 67 24–29.

[B31] Zellner D. A. (2013). Color-odor interactions. a review and model. *Chemosens. Percept.* 6 155–169. 10.1007/s12078-013-9154-z

