# Supplementary material for: Effects of Coloring Food Images on the Propensity to Eat: A Placebo Approach With Color Suggestions
Source: Front Psychol. 2020 Oct 29;11:589826. doi: 10.3389/fpsyg.2020.589826 (PMC7658407; doi:10.3389/fpsyg.2020.589826)
Supplement: Supplementary Table 1 — Descriptive Statistics (means and standard deviations (M, SD) for the participants in the non-suggestion condition and the suggestion condition in Experiment 1 and Experiment 2. [file Table_1.pdf]

Supplementary Table 1: Descriptive Statistics (means and standard deviations (M, SD) for the participants in the non-suggestion condition and the suggestion condition in experiment 1 and experiment 2.

	**Experiment 1**		
	**condition**	***M***	***SD***
**Age**	No-suggestion	21.64	2.93
	Suggestion	22.92	3.47
**BMI**	No-suggestion	21.91	2.83
	Suggestion	21.79	2.97
**Last meal**	No-suggestion	2.70	3.43
	Suggestion	3.07	3.04
**Hunger level**	No-suggestion	2.78	2.07
	Suggestion	3.14	2.21
		N	
	No-suggestion	99	
	Suggestion	118	

	**Experiment 2**		
	**condition**	***M***	***SD***
**Age**	No-suggestion	22.21	3.33
	Suggestion	23.26	3.43
**BMI**	No-suggestion	21.45	2.65
	Suggestion	21.84	3.23
**Last meal**	No-suggestion	3.44	3.60
	Suggestion	3.89	3.93
**Hunger level**	No-suggestion	3.09	2.38
	Suggestion	3.11	2.34
		N	
	No-suggestion	118	
	Suggestion	113	

Footnote: last meal = “When was the last time you ate (in hours)?”; hunger = “How hungry are you at the moment?” (1 = “not hungry at all”; 9 = ”very hungry”); BMI = “Body mass index”
